# Common genetic variants on chromosome 9p21 are associated with myocardial infarction and type 2 diabetes in an Italian population

**DOI:** 10.1186/1471-2350-11-60

**Published:** 2010-04-19

**Authors:** Francesca Gori, Claudia Specchia, Silvia Pietri, Luisa Crociati, Simona Barlera, Monica Franciosi, Antonio Nicolucci, Stefano Signorini, Paolo Brambilla, Maria Grazia Franzosi

**Affiliations:** 1 Department of Cardiovascular Research, "Mario Negri" Institute for Pharmacological Research, Milano, Italy; 2 Department of Biomedical Sciences and Biotechnologies, University of Brescia, Brescia, Italy; 3 Consorzio Mario Negri Sud, Santa Maria Imbaro, Italy; 4 University Department of Laboratory Medicine, Hospital of Desio, Milano, Italy; 5 University of Milano-Bicocca, Medical School DMS, Milano, Italy

## Abstract

**Background:**

A genomic region on chromosome 9p21 has been identified as closely associated with increased susceptibility to coronary artery disease (CAD) and to type 2 diabetes (T2D) although the evidence suggests that the genetic variants within chromosome 9p21 that contribute to CAD are different from those that contribute to T2D.

We carried out an association case-control study in an Italian population to test the association between two single nucleotide polymorphisms (SNPs) on the 9p21 locus, rs2891168 and rs10811661, previously reported by the PROCARDIS study, and respectively myocardial infarction (MI) and T2D. Our aim was to confirm the previous findings on a larger sample and to verify the independence of their susceptibility effects: rs2891168 associated with MI but not with T2D and rs10811661 associated with T2D but not with MI.

**Methods:**

Genomic DNA samples of 2407 Italians with T2D (602 patients), who had had a recent MI (600), or had both diseases (600) and healthy controls (605) were genotyped for the two SNPs. The genotypes were determined by allelic discrimination using a fluorescent-based TaqMan assay.

**Results:**

SNP rs2891168 was associated with MI, but not with T2D and the G-allele odds ratio (OR) was 1.20 (95% CI 1.02-1.41); SNP rs10811661 was associated with T2D, but not with MI, and the T-allele OR was 1.27 (95% CI 1.04-1.55). ORs estimates from the present study and the PROCARDIS study were pooled and confirmed the previous findings, with greater precision.

**Conclusions:**

Our replication study showed that rs2891168 and rs10811661 are independently associated respectively with MI and T2D in an Italian population. Pooling our results with those reported by the PROCARDIS group, we also obtained a significant result of association with diabetes for rs10811661 in the European population.

## Background

Recent genome-wide association studies (GWAS) have identified a genomic region on chromosome 9, approximately 22 million base pairs from the 9p telomere, closely associated with increased susceptibility to coronary artery disease (CAD) [1-4]. This region maps near two well-characterized tumor suppressor genes, CDKN2A and CDKN2B, encoding respectively proteins p16 ^INK4a ^and p15 ^INK4b^, involved in the regulation of cell proliferation, cell aging and apoptosis [5], mechanisms that have a critical role in atherosclerosis [6]. Protein p16^INK4a ^inhibits cyclin-dependent kinase 4(CDK4) and is a strong regulator of pancreatic beta cell replication [7-9]. The same region has also recently been associated with abdominal aortic and intracranial aneurysms [10].

Three recent studies found increased susceptibility to type 2 diabetes (T2D) for carriers of single nucleotide polymorphism (SNP) rs10811661 mapping the chromosome 9p21 [11-13]. The same allelic variant showed the strongest and most consistent association in a meta-analysis of T2D GWAS [12] (Table 1).

**Table 1:** Association of rs2891168 with coronary artery disease (CAD) and rs10811661 with type 2 diabetes (T2D) in different populations.

SNP	Risk allele	Risk allele frequency	Study population (no. cases)	OR^§^ (95% CI)	P-value	Genetic approach	Ref.*
rs2891168	G	0.59	Europeans (4251)	1.29 (1.20-1.38)	6 × 10^-13^	Case-control study	[15]
rs10811661	T	0.85	Finns (2314)	1.20 (1.07-1.36)	2.2 × 10^-3^	GWAS	[11]
		0.82	UK (5681)	1.19 (1.11-1.28)	4.9 × 10^-7^	Meta-analysis of GWAS	[12]
		0.83	Scandinavians Poles, US (6529)	1.20 (1.12-1.28)	5.4 × 10^-8^	GWAS	[13]
		0.86	Dutch (9132)	1.30 (1.16-1.47)	1 × 10^-5^	Validation of GWAS	[24]
		0.56	Chinese Hans (1302)	1.31 (1.12-1.54)	1 × 10^-3^	Replication study	[25]

* Ref: reference

^§ ^OR: Odds Ratio

T2D has long been recognized as a major risk factor for atherosclerosis, and therefore for CAD, although their temporal and etiological relationships are not clear: CAD usually follows diabetes mellitus, though it can sometimes precede it. Doria et al. showed that the CAD risk associated with a 9p21 variant was higher in type 2 diabetic patients with poor glycemic control [14].

The presence of several different SNPs within chromosome 9p21 associated with such tightly related diseases prompted us to investigate a shared susceptibility variant, although the evidence suggests that different genetic variants within chromosome 9p21 contribute to CAD and T2D. The PROCARDIS Consortium's results showed that two different SNPs, rs2891168 and rs10811661, both in the 9p21 region although rs2891168 belongs to the CAD-associated locus while rs10811661 is localized outside, are associated with CAD and diabetes respectively, and the two susceptibility effects are independent [15]. However, there are several reasons why the PROCARDIS results cannot be regarded as conclusive. Most importantly, the study was not conceived, and did not have sufficient statistical power to answer this question as the number of diabetic patients without CAD was quite small. To confirm and replicate these results, a larger sample was deemed necessary.

Therefore we carried out a case-control association study in a population of Italian patients with T2D only, myocardial infarction (MI) only, or both diseases, to test the susceptibility effects of rs2891168 and rs10811661. Our aim was to confirm previous findings on a larger sample and to verify whether the effects of these two SNPs on susceptibility to MI and T2D were actually independent: rs2891168 associated with MI but not with T2D and rs10811661 associated with T2D but not with MI.

## Methods

### Study population

The study population consists of 2407 unrelated Italians, 1739 men and 668 women.

We enrolled all the patients (602) with T2D who had a blood sample available among those in the IGLOO cohort study (Impaired Glucose intolerance & Long-term Outcomes Observational Study) [16]. The other case groups of 600 MI only and 600 MI&T2D were selected at random from the GISSI-Prevenzione study (GISSI-P) [17]. GISSI-P and IGLOO are both multicentre Italian studies approved by the local ethics committees of the participating hospitals (see references 16,17 for the complete list). Written informed consent to participate in the study, including blood sampling, was obtained for each subject.

The characteristics of the participants have been described previously [16,17]. In brief, GISSI-P patients were selected on the basis of a clinical diagnosis of recent (< = 3 months) MI, with no limits of age and distinction of sex. The IGLOO population comprised men and women aged between 55 and 75 years, with no history of cardiovascular events such as angina and MI but with one or more cardiovascular risk factors. All patients were referred to a diabetes outpatient clinic for an oral glucose tolerance test, with determination of venous plasma glucose, fasting and 2 hours after the ingestion of 75 g glucose. Those recruited for the present study all had T2D.

Major details of the study design, eligibility criteria, and IGLOO and GISSI-P results are reported elsewhere [16,17].

A group of 605 unmatched Italian controls was recruited among blood donors listed with AVIS (the Italian blood donors' association). Donors have regular routine check-ups of their state of health. The following inclusion criteria were used: age < = 65 years, no history of diabetes, no CAD and no first-degree relatives with CAD. Therefore, controls were expected to be at a very low risk of cardiovascular events and T2D.

Patients in the T2D group were older but the other groups were close in age (Table 2).

**Table 2:** Main details of the study groups: sample size, sex and age.

group	No. of subjects	Women (%)	Age (yrs) mean (SD*)
T2D^§^	602	43.2	62.4 (7.3)
MI^†^	600	20.8	57.5 (6.5)
MI&T2D	600	28.2	58.2 (7.5)
Controls	605	18.8	56.6 (6.5)

Total	2407	27.7	58.7 (7.3)

*SD: standard deviation;

^§^T2D: type 2 diabetes;

^†^MI: myocardial infarction.

The studied population can be considered representative of Italians from all regions and homogeneous in terms of genetic constitution since subjects derived from the previous studies and controls are all white Italians, of European ancestry, without the possibility to isolate any subpopulations based on ethnicity, differences in disease prevalence and/or risk allele frequencies among groups.

### SNP selection

Two previously reported susceptibility SNPs on chromosome 9p21 [15] (rs2891168 and rs10811661) were selected for genotyping in the present study and are described in Table 3.

**Table 3:** Characteristics of the SNPs rs2891168 and rs10811661 in the study population.

SNP	Position (bp)	HWE* χ^2 ^test p-value	Risk allele	Risk allele frequency
rs2891168	21921500	0.81	G	0.59
rs10811661	22124094	0.80	T	0.80

* HWE: Hardy-Weinberg Equilibrium

We selected these genetic variants in view of our aim to replicate the PROCARDIS investigators' analysis who selected these out of 11 literature SNPs initially considered. Using an entry P-value threshold of 0.01, SNP rs2891168 was selected as it showed the strongest association with CAD (P = 6 × 10^-13^) in their population, with a per-G allele odds ratio (OR) of 1.29 (95% CI 1.20-1.38) [15]. rs10811661 has been identified through the recent GWAS [11-13] and showed the most consistent association in a meta-analysis of T2D GWAS [12].

### Genotyping

DNA was extracted from frozen EDTA-whole blood using a salting-out procedure [18]. All polymerase chain reactions (PCRs) were done in a volume of 5 μl containing TaqMan universal PCR Master Mix, specific TaqMan^® ^SNP Genotyping Assays, purchased from Applied Biosystems (ABI) (Foster City, CA, USA), and 10 ng of genomic DNA, according to the manufacturer's instructions. The 7900 Real-Time PCR System (ABI) was used for SNP genotyping. The fluorescent data files for each plate were analyzed using Sequence Detection System version 3.2 (ABI). To ensure the quality of automatic allele calling, all samples were analyzed in two replicates and the concordance rate was 100%. No genotype data were missing.

### Statistical analysis

Hardy-Weinberg (HW) equilibrium was tested using the genhw STATA package. Additive and non-additive effects were modeled by comparing an additive model in terms of logit, i.e. a model of allelic association, and a model of genotype association, by the likelihood ratio test. Association analyses were done between the presence of the SNP rs2891168 G-allele and the SNP rs10811661 T-allele and MI, T2D and MI&T2D case groups using unconditional multinomial logistic regression. ORs and 95% confidence intervals (CI) were calculated comparing case groups with the controls. Homogeneity of risks among case groups was assessed using a Wald test with one degree of freedom [19]. ORs estimates in the present study and in the PROCARDIS study [15] were pooled using a fixed-effects model.

The QUANTO software was used for power calculation on pooled data [20] and STATA 9.0 for the statistical analysis.

## Results

Both SNPs were in HW equilibrium among controls (p ≥ 0.3). Risk allele frequencies in the study population were in agreement with published data [15]. Single marker tests supported an additive genetic model on the logit scale (p > 0.10), consistent with an allelic association model.

**Figure 1:**
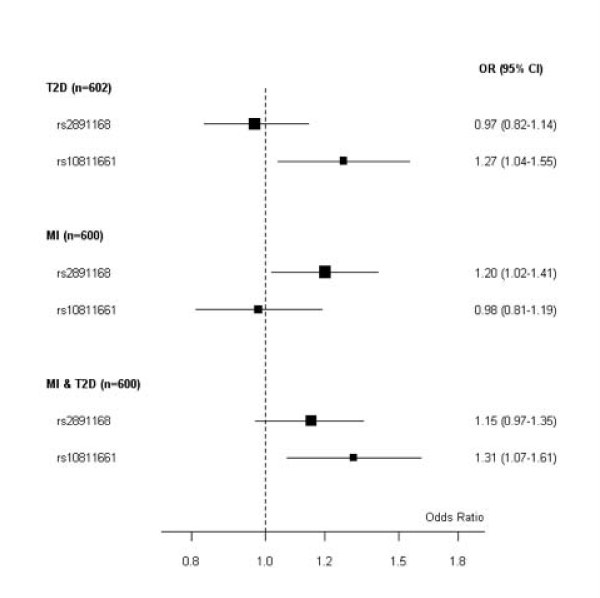
**rs2891168 association with MI and rs10811661 with T2D in the Italian sample**. Solid squares are centered on the ORs and scaled in proportion to the inverse variance of the estimates with 95% CI (horizontal bar) for groups, comparing MI with or without a clinical history of diabetes. The reference group is non-MI, non-diabetics (605). The number of individuals in each group (n) is shown.

In unconditional multinomial logistic regression modeling additive genetic effects, SNP rs2891168 was associated with MI (p = 0.03) and not with T2D, and the G-allele OR was 1.20 (95%CI 1.02-1.41). SNP rs10811661 was associated with T2D (p = 0.02) and not with MI, and the T-allele OR was 1.27 (95%CI 1.04-1.55). ORs and 95% CIs calculated by comparing cases (T2D, MI, MI&T2D) with controls are reported in Figure 1.

Comparing the homogeneity of genetic risks in the diagnostic groups, the rs2891168 G-allele OR of the MI&T2D group was significantly different from the T2D group (p = 0.04), while the rs10811661 T-allele OR of the MI&T2D group was significantly different from the MI group (p = 0.005). These results indicated that SNP rs2891168 was associated with MI, SNP rs10811661 with T2D, and confirmed that the two susceptibility variants are independently associated with the two separate diseases in our Italian population.

**Figure 2:**
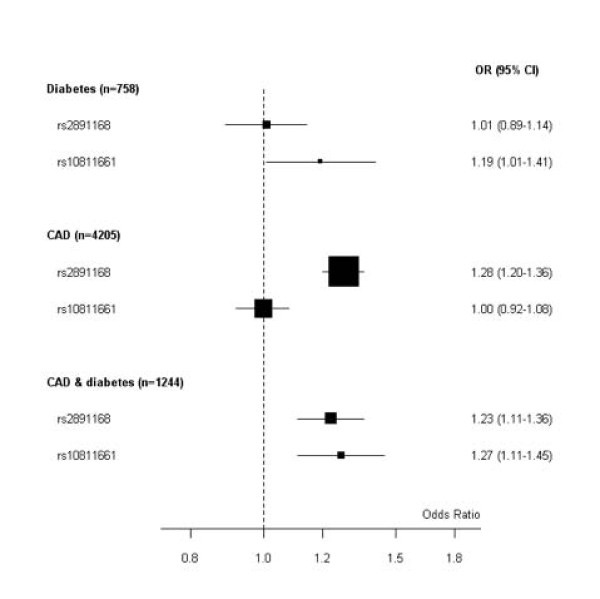
**rs2891168 association with CAD and rs10811661 with diabetes: pooled estimates with PROCARDIS data**. Solid squares are centered on the ORs and scaled in proportion to the inverse variance of the estimates with 95% CI (horizontal bar) for groups comparing CAD with or without a clinical history of diabetes. The reference group is non-CAD, non-diabetics (4910). The number of pooled individuals in each group (n) is shown.

Pooled ORs were calculated using the PROCARDIS results and are reported in Figure 2.

SNP rs2891168 was confirmed as associated with CAD (p < 0.0001) with a G-allele OR of 1.28 (95% CI 1.20-1.36), and SNP rs10811661 was associated with T2D (p = 0.04) with a T-allele OR of 1.19 (95%CI 1.01-1.41).

Given these allele frequencies and assuming a 5% prevalence of diabetes [21], and a type 1 error rate of 0.05, a post-hoc power calculation indicated that our sample of Italian T2D cases (602) pooled with the PROCARDIS diabetics (156) had 80% power to detect an association in terms of an OR of 1.30 between the rs10811661 T variant and diabetes.

## Discussion

Estimates of genetic effect size from genome-wide screens are frequently biased [22] and more precise estimates can be obtained in independent replication cohorts. In the PROCARDIS study the magnitude of the susceptibility effect for CAD (OR = 1.29; 95% CI: 1.20-1.38) was very similar to previous GWAS [15]. The main finding of the present study is therefore its ability to replicate the analysis done in the European PROCARDIS population on an independent cohort of Italians, with comparable inclusion criteria.

The ORs for the susceptibility effects of rs2891168 and rs10811661 on MI and T2D (Figure 1) are in line with previous evidence that these genetic signals are independently associated with the two diseases. CAD susceptibility conferred by rs2891168 was strongly proved in the PROCARDIS population. Its susceptibility effect on MI (OR = 1.20; 95%CI 1.02-1.41) in the Italian population is less strong. However, PROCARDIS patients had documented diagnoses of MI, symptomatic acute coronary syndrome, intervention for coronary revascularization, or chronic stable angina, the four major diagnostic outcomes [23] for a heterogeneous disease like CAD. There is considerable overlap between these outcomes, yet their underlying pathophysiology differs significantly. In this respect the Italian patients considered in the present study are more homogeneous: they are surely CAD patients but all had a clinically diagnosed MI.

By contrast, there was no conclusive evidence of an association between rs10811661 and diabetes risk in the PROCARDIS analysis, probably because the diabetic group was too small to produce reliable results. A limitation the PROCARDIS authors mentioned is that the diabetic population included individuals with both types 1 and 2 diabetes without the possibility to distinguish them. In the present study the diabetic patients all had T2D, so it is important to note that rs10811661 is a genetic risk factor for T2D in the Italian population (OR = 1.27; 95%CI 1.04-1.55), as has been reported so far for several European populations [11,12,24] and for the Han Chinese [25], with an average associated OR of 1.25 for the risk allele variant, although the East Asians have a lower prevalence of diabetes and different risk allele frequency from Europeans [26].

Pooling our data and the PROCARDIS data, thus increasing the sample size, we confirmed the association of rs2891168 with CAD and, most importantly, we found a significant association with diabetes for rs10811661 in the European population. Consistent results in the two studies and the greater precision of the pooled estimates support the association and confirm the hypothesis on this larger sample.

According to the PROCARDIS data analysis [15], the association model between SNPs and case groups was not adjusted for confounders. Actually they tested whether the susceptibility between CAD and rs2891168 changed significantly in subgroups of CAD patients (regular smoker, sex, age, obesity, diabetes and hypertension); none of these potential confounders affected the associations. In order to pool our data with those of the PROCARDIS study we used the same statistical approach. Moreover, available covariates are intermediate phenotypes for the outcome of interest and their inclusion as exposures may reduce the effect of overlapping genetic factors.

Sometimes replication studies fail to confirm initial findings because of substantial differences between study populations and population specificity that may consist in differences in linkage disequilibrium (LD) block, population-specific interactions between genes, and epigenetic modifications. Therefore, the fact that the present study replicated a significant evidence of association in an independent cohort of Italians helps clarify the genetic component's contribution in different populations for multifactorial diseases like MI and T2D, so it can be considered a step forward to producing trustworthy results.

Despite the many GWAS done in the last few years, and the many genetic factors identified, the precise genetic background to complex human diseases such as CAD and T2D is still not clear. There is strong evidence of associations between common variants within chromosome 9p21 and the risk of CAD and T2D, but so far the biological function of most of them and how they are linked with the clinical phenotype is still not known. Further physiological and functional studies are needed to clarify the molecular mechanisms and pathways underlying the associations with CAD or T2D. These could help identify biological targets for the prevention or treatment of these common diseases and may establish whether certain allelic variants have any effects on other genes, for instance CDKN2A and CDKN2B, since they are the closest to the 9p21 CAD and T2D loci.

Helgadottir et al. reported that chromosome 9p21 is associated not only with MI but also with an increased risk to develop abdominal aortic and intracranial aneurysms [10]. Björck et al. also found an association between genetic variation on chromosome 9p21.3 and arterial stiffness [27]. These findings suggest that this locus is not directly and/or exclusively involved in the pathogenesis of MI but may play an important role in the integrity of the vessel wall, thus influencing a broad range of cardiovascular disorders.

## Conclusions

In summary, we report that SNPs rs2891168 and rs10811661 are independently associated respectively with MI and T2D in an Italian population. Combining our results with those reported by the PROCARDIS group, we found a significant association with diabetes for rs10811661 in the European population.

## Competing interests

The authors declare that they have no competing interests.

## Authors' contributions

MGF, FG, SP, LC, CS and SB conceived the study and participated in its design and coordination. FG carried out the genotyping assay, analyzed and interpreted the data, and drafted the manuscript. CS and SB did the statistical analysis; CS was also involved in drafting the manuscript. SP collected the controls, helped interpret the data and carried out the genotyping. LC was the data manager. MF and AN recruited the T2D patients. SS and PB are responsible for the SiBioC-GISSI-Prevenzione biobank. MGF was involved in the critical revision of the manuscript for major intellectual content. All authors read and approved the final manuscript.

## Authors' informations

FG, Biotech.D., Claudia Specchia, Mat.Sci.D., Ph.D., Silvia Pietri, Lab.Techn., Luisa Crociati, Biol.Sci.D., Simona Barlera, Sci.Pol.D., MSc., Monica Franciosi, MSc (Biol), Antonio Nicolucci, MD, Stefano Signorini, MD, Paolo Brambilla, MD, Maria Grazia Franzosi, Biol.Sci.D. Head of the Cardiovascular Research Department, "Mario Negri" Institute for Pharmacological Research.

## Pre-publication history

The pre-publication history for this paper can be accessed here:

http://www.biomedcentral.com/1471-2350/11/60/prepub
